# Supplementary material for: The inclusion of blastomeres into the inner cell mass in early-stage human embryos depends on the sequence of cell cleavages during the fourth division
Source: PLoS One. 2020 Oct 19;15(10):e0240936. doi: 10.1371/journal.pone.0240936 (PMC7571684; doi:10.1371/journal.pone.0240936)
Supplement: Attachment — Submitted filename: Response to Reviewers 20200928.docx [file pone.0240936.s002.docx]

**Response to Reviewer 1**

We appreciate the positive feedback and comments.

Comment 1: The observation is interesting, however, the mechanism or biological relevance remained elusive.

Answer to comment 1:

As a very recent paper published from Nature provided significant insight into our findings, we discussed the possible mechanism. This mechanism can be considered in the context in the light of both the Nature publication and our findings in this manuscript. The mechanism is explained in the revised manuscript with track changes (Page11-12 Line189-195 yellow highlighted).

Comment 2: Can authors comment on blastocyst quality and pregnancy outcome from embryos that had ICM from the blastomeres which cleaved first to fourth vs. the blastomeres which cleaved fifth to eight?

Answer to the comment 2:

We have calculated the blastocyst quality and pregnancy outcomes of embryos that had ICM from the blastomeres which cleaved first to fourth vs. the blastomeres which cleaved fifth to eighth. This data has been explained and discussed in the revised manuscript with track changes (Page7 Line 116-122, Page10 Line159-162 yellow highlighted).
